# A comprehensive cuproptosis score and associated gene signatures reveal prognostic and immunological features of idiopathic pulmonary fibrosis

**DOI:** 10.3389/fimmu.2023.1268141

**Published:** 2023-11-14

**Authors:** Chuanqing Jing, Rong Fu, Xue Liu, Guodong Zang, Xue Zhu, Can Wang, Wei Zhang

**Affiliations:** ^1^ Clinical Department of Integrated Traditional Chinese and Western Medicine, The First Clinical Medical College of Shandong University of Traditional Chinese Medicine, Jinan, China; ^2^ Department of Respiratory and Critical Care Medicine, Affiliated Hospital of Shandong University of Chinese Medicine, Jinan, China

**Keywords:** cuproptosis, IPF, prognosis, immunity, molecular subtype, gene signatures

## Abstract

**Background:**

Cuproptosis, the most recently identified and regulated cell death, depends on copper ions *in vivo*. Copper regulates the pathogenesis of Idiopathic pulmonary fibrosis (IPF), but the mechanism of action underlying cuproptosis in IPF remains unclear.

**Methods:**

We identified three cuproptosis patterns based on ten cuproptosis-related genes using unsupervised consensus clustering. We quantified these patterns using a PCA algorithm to construct a cuproptosis score. ssGSEA and the Cibersort algorithm assessed the immune profile of IPF patients. GSEA and GSVA were used to analyze the functional differences in different molecular patterns. Drug susceptibility prediction based on cuproptosis scores and meaningful gene markers was eventually screened in combination with external public data sets,*in vitro* experiments and our cases.

**Results:**

Of the three types of cuproptosis-related clusters identified in the study, patients in the clusterA, geneclusterB, and score-high groups showed improved prognoses. Moreover, each cluster exhibited differential immune characteristics, with the subtype showing a poorer prognosis associated with an immune overreaction. Cuproptosis score can be an independent risk factor for predicting the prognosis of IPF patients. GSEA showed a significant functional correlation between the score and cuproptosis. The genes *AKAP9, ANK3, C6orf106, LYRM7*, and *MBNL1*, were identified as prognostic-related signatures in IPF patients. The functional role of immune regulation in IPF was further explored by correlating essential genes with immune factors. Also, the nomogram constructed by cumulative information from gene markers and cuproptosis score showed reliable clinical application.

**Conclusions:**

Cuproptosis patterns differ significantly in the prognosis and immune characteristics of IPF patients. The cuproptosis score and five gene signatures can provide a reliable reference in the prognosis and diagnosis of IPF.

## Introduction

Idiopathic pulmonary fibrosis (IPF) is a rare, chronic, progressive, fibrotic, interstitial lung disease with unknown etiology and pathogenesis (1). It mainly affects the middle-aged and elderly population with a poor prognosis. It is clinically characterized by progressive scarring or fibrosis of the interstitial lung, leading to a gradual decline in lung function and eventual death (2, 3). The prevalence of IPF in the general population ranges from 2/100,000 to 29/100,000, with a reported male-to-female ratio of nearly 2:1 and a median survival of only 2-3 years after diagnosis (4). The pathogenesis of IPF is primarily associated with excessive extracellular matrix (ECM) deposition, alveolar epithelial damage, endoplasmic reticulum stress, and immune regulation imbalance (5–7). Clinical treatment of IPF is limited; two anti-fibrotic drugs, pirfenidone and nintedanib, slow the progression of pulmonary fibrosis but fail to entirely cure it (8). Several prognostic assessment systems have been established based on blood, bronchoalveolar lavage fluid, and clinical indicators (9–11). However, individual prognostic markers and unidimensional analysis lack reliability for predicting the prognosis of this heterogeneous disease. Hence, it is clinically crucial to identify the molecular subtypes of IPF and develop multivariate predictive models.

Cuproptosis is a novel regulated form of cell death characterized by excessive accumulation of copper ions in the body, leading to cell death (12). Copper ions induce cell death by binding directly to crucial enzymes of the tricarboxylic acid (TCA) cycle (DLAT, GCSH, DLST, DBT), which undergo protein-lipid acylation to form aggregates, leading to the loss of iron-sulfur cluster proteins, thereby causing proteotoxic stress. The study further confirmed how cells dependent on mitochondrial respiration are more sensitive to copper ion carriers and that inhibition of the electron transport chain complex and pyruvate carriers attenuated cuproptosis (13, 14). Programmed cell death such as autophagy and apoptosis have been shown to be closely related to IPF, but copper death has been limitedly studied in IPF (15, 16).In addition, it has been shown that copper ion carriers and copper transporter proteins are associated with epithelial mesenchymal transition (EMT), angiogenesis,which are closely related to the pathogenesis of pulmonary fibrosis (17, 18). The disease tissue is often hypoxic during the development of fibrotic disease affecting the body’s energy metabolism and leading to obstruction of the tricarboxylic acid cycle (19, 20). Such evidence suggested that there may be a potential link between cuproptosis and IPF.

In this study, we constructed three subtypes of cuproptosis based on cuproptosis-related regulatory genes. We also proposed for the first time the need to assess the prognostic characteristics of each IPF patient using the cuproptosis score, further screening the gene markers and exploring the underlying immune-related mechanisms of IPF. Finally, we constructed a nomogram of gene markers and cuproptosis scores to establish a reference for clinical prognosis.

## Materials and methods

### Data preparation and processing

The datasets used in this paper (GSE27957, GSE28042, GSE38958) were downloaded from the Gene Expression Omnibus database (GEO, https://www.ncbi.nlm.nih.gov/geo/). The GSE27957 (21) and GSE28042 (21) datasets were used as the discovery cohort for this study. The GSE27957 dataset is based on the GPL5175 platform (Affymetrix Human Exon 1.0 ST Array [transcript (gene) version]) and includes samples from 45 idiopathic pulmonary fibrosis patients (IPF) with peripheral blood mononuclear cells (PBMC). The GSE28042 dataset is based on the GPL6480 platform (Agilent-014850 Whole Human Genome Microarray 4x44K G4112F), and the sequencing samples include PBMC from 75 IPF patients. Survival for patients in both datasets was calculated with transplant-free survival (TFS) information, considering transplantation or death as the endpoint event. The GSE38958 dataset based on the GPL5175 (Affymetrix Human Exon 1.0 ST Array) included PBMC sequencing data from 65 IPF patients and 45 healthy individuals and was used as the validation cohort (22). The dataset was merged using the “limma” package (Version 3.50.3) (23)and the “SVA” package (Version 3.42.0) (24) to eliminate batch effects. Principal component analysis (PCA) was used to determine the degree of convergence between patients in the two datasets. Cuproptosis-related genes were derived from a recent study by Peter Tsvetkov et al. (13).

### Identification of prognostic features of cuproptosis-related genes

The literature review shows ten cuproptosis-related genes (*FDX1, LIAS, LIPT1, DLD, DLAT, PDHA1, PDHB, MTF1, GLS, CDKN2A*) were identified in this study. Correlation and univariate COX regression analysis of the ten genes were performed using the software package “igraph” (Version 1.3.5), the “psych” program package (Version 2.2.9), and the “reshape2” program package (Version 1.4.4). The threshold of correlation between the genes was set at *p*<0.0001. At the same time, survival analysis was performed using the “survminer” program package (Version 0.4.9) and “survival” program package (Version 3.4-0) to identify the prognostic features of the ten genes.

### Consensus clustering analysis based on cuproptosis-related genes

The “ConsensusClusterPlus” software package (25) was used for the unsupervised clustering of data from 120 IPF patients based on their mRNA expression profiles of the ten cuproptosis-related genes. The patients were divided into Cluster 1 and Cluster 2 groups based on optimal k values. Survival analysis by the “survminer” package and “survival” package was used for generating the Kaplan-Meier (K-M) survival curves. The “limma” package (Version 3.50.3) (23) was then used to identify differences in cuproptosis-related gene expression between patients with the two patterns, and “pheatmap” (Version 1.0.12) was used to demonstrate the relationship between clinical features, cuproptosis-related gene expression, and subtypes.

### Gene set variance analysis

The hallmark (h.a ll. 7.5.1. Symbols), KEGG (c2. Cp. KEGG. 7.5.1. Symbols), and Reactome (c2. Cp. Reactome. 7.5.1. Symbols) pathway gene sets were downloaded from the MsigDb database (https://www.gsea-msigdb.org/gsea/msigdb). The R package GSVA (Version 2.11) (26) was used to score the pathways. The “limma” R package (version 3.50.3.1) (23) was used to determine the differentially enriched or depleted pathways and their biological functions. We compared the pathway enrichment differences between two molecular subtypes of cuproptosis. |log2 FC | > 0.1 and adj. *p*-value < 0.05 has been considered as significant enrichment. The R package “pheatmap” (Version 1.0.12) was used to draw the heatmap.

### Differential gene expression and functional enrichment analysis of two cuproptosis molecular patterns

Principal component analysis (PCA) was used to observe the distribution of patients falling between the two cuproptosis molecular patterns. Then, differential gene expression analysis was performed using the “limma” program package (23) for C1 and C2 subtypes to obtain the differentially expressed genes (DEGs). A total of 178 DEGs were obtained using |log2 FC|>0.5 and *p*<0.05 as thresholds to screen for DEGs.

Further, we performed a gene ontology (GO), and kyoto encyclopedia of genes and genomes (KEGG) condensed analysis of all the DEGs using the “ClusterProfiler” R package (Version 4.2.2) (27, 28).

### Identification of clusters and prognostic features associated with differentially expressed genes between the two cuproptosis molecular patterns

The 178 DEGs were subjected to univariate Cox regression analysis, and a threshold of *p*-value < 0.05 was set that helped us identify 12 cuproptosis differentially expressed genes (Cu-DEGs) with prognostic significance. Unsupervised consensus cluster analysis was performed on 120 IPF patients divided into cluster 1 and cluster 2 based on the expression of the Cu-DEGs. Next, survival analysis was performed using the “survminer” program package (Version 0.4.9) and the “survival” program package (Version 3.4-0) for both types of patients and represented using Kaplan-Meier (KM) curves to demonstrate their prognostic features. The “limma” package (Version 3.50.3) (23) was used to determine the expression levels of the Cu-DEGs between the two types of patients, and the “pheatmap” package (Version 1.0.12) was used to show the relationship between clinical features, Cu-DEGs expression level, and cuproptosis gene clusters.

### Cuproptosis score and prognostic analysis

Based on the 12 differential genes to screen the cuproptosis features related to typing, those positively or negatively correlated with the differential genes respectively were designated as AB feature genes, which were downscaled using the Boruta algorithm, and the transcriptome patient information was extracted for the scoring using the method of Principal Component Analysis PCA, with PC1A denoting the first component of feature A, and PC1B denoting the first component of feature B, which was calculated using the formula as follows: cuproptosis score = ∑PC1A - ∑PC1B. The optimal cutoff value was determined by Kruskal-Wallis and divided into high and low groups, followed by survival analysis of patients in both groups. (29, 30).We used the “ggalluvial” R program package (Version 0.12.3) for Sankey mapping to show the relationship between the clusters, gene clusters, cuproptosis score, and prognostic status. The differences in the cuproptosis scores between clusters, gene clusters, and variable survival states of patients were assessed using the Kruskal-Wallis test, and the proportion of patients with varying states of survival in the two scores was plotted. We further analyzed the diagnostic effectiveness of the cuproptosis scoring system using the “pROC” package (Version 1.18.0) (31).

### Validation of external datasets

Data from GSE38958 were analyzed for differences using the “limma” package (Version 3.50.3) (23), setting cutoff values of *p*<0.05 and |log2 FC|>0. Genes that were differentially expressed in IPF patients and healthy individuals were screened and visualized with volcano plots and heat maps. Next, differential expression analysis of the 12 Cu-DEGs was performed, and seven genes (*AKAP9, ANK3, C6orf106, LYRM7, MBNL1, NPCDR1, LIG4)* were differentially expressed in the GSE38958 validation set. Volcano, heat, and box plots were also plotted to visualize the differences in the expression of these seven genes in healthy patients compared to IPF patients in the GSE38958 dataset.

### Identification of key genes

Among the seven genes mentioned above, the expression of six genes that consistently correlated with prognosis prediction included AKAP9, ANK3, C6orf106, LYRM7, MBNL1, and NPCDR1. Correlation analysis was performed on each of these six genes to demonstrate the expression of the 50 genes most positively associated with each gene to assess the impact of the critical genes on disease. In parallel, we performed a spearman analysis of the correlations between the six essential genes and represented them using heat maps using “ggplot2”. A t-test was then used to correlate the vital genes and cuproptosis scores to evaluate the relationship between critical genes and cuproptosis scores.We also further analyzed the relationship between these genes and fibrosis marker genes such as cytokines and extracellular matrix genes (interleukin 4, collagen I, α-smooth muscle actin,fibronectin 1) and visualized them in a heat map.

### Drug sensitivity prediction

The “pRRophetic” R package (32) was used to predict the IC_50_ values of each sample for multiple drugs, and the “limma” package (Version 3.50.3) (23) was applied to compare the differences between the high and low fractional values, with lower IC_50_ values indicating greater sensitivity to the drugs. We screened for chemical agents whose targets were associated with the pathogenesis of pulmonary fibrosis and for which no cases of drug-induced interstitial pneumonia were reported. We visualized the results with the “ggplot2” program package (Version 3.3.6).

### Immuno-infiltration analysis

A set of marker genes for immune-related cells and functions was obtained through a literature search (33). After removing immune cells absent from the blood samples, this study used the “single sample gene set enrichment analysis” (ssGSEA) algorithm to calculate the functional scores of immune cells and immune functions for each IPF sample based on the expressed gene signatures using the GSVA program package. We then compared their expression levels in different subtypes. Moreover, we analyzed the correlation between immune cells, immune function, and cuproptosis score using the t-test. In addition, we calculated the content of 16 immune cell types in patients in the training set using the CiberSort algorithm (34). We downloaded the list of marker genes for immune modulators from the TISIDB database (http://cis.hku.hk/TISIDB/). According to the expression levels of six essential genes, the correlation analysis between them and the expression of immune cells and immune functions obtained by the ssGSEA algorithm was conducted to observe the function characteristics of IPF in immunity. Next, the “corrplot” package (Version 0.92) was used to visualize the correlation between the immune cell content, the expression level of immune modulators, and critical genes obtained by the Cibersort algorithm to get the immune regulation mode of IPF.

### Gene set enrichment analysis

Gene set enrichment between copper-mediated death high and low-scoring models were obtained by setting adj *p*-value<0.05 and false discovery rate (FDR) <0.05 for significantly enriched. The “c2.cp.v7.2.symbols.gmt [Curated]” was used as a gene set for GSEA to observe signaling mechanisms between different scores (35). In addition to correlation analysis, this study also carried out single gene GSEA analysis using the R package “clusterProfiler” for the six selected vital genes. The 50 genes most closely related to each essential gene were selected as input values, and *p*<0.05 was considered significant enrichment to identify the signaling pathways related to essential genes (27).

### Single-cell analysis

In order to further understand the role of key genes in IPF, the GSE132771 dataset (5) was downloaded from the GEO database, including single-cell sequencing samples of lung tissues from three patients with IPF and three normal subjects, and the data were subjected to quality control and normalization analysis, with the following criteria: minGene=200, maxGene=4000, pctMT=10. Sample batches were removed using the “Harmony” program (Version 1.1.1). Cell annotation was performed using the “SingleR” package (Version 1.8.1). Subsequently, the “reshape2” (1.4.4) and “ggplot2” packages (3.4.3) were used to map the proportions of each cell subpopulation and the subcellular localization of key genes.

### *In vitro* study

Modeling was performed using human embryonic lung fibroblasts, the MRC-5 cell line was purchased from Procell manufacturer, and the cells were cultured in MEM (with NEAA) medium containing 10% FBS as well as 1% P/S double antibody at 37°C in a 5% CO2 air environment. The cells were stimulated with 10ng/ml TGF-β1 for 48h for model construction of pulmonary fibrosis.Total RNA and protein were collected for qPCR and protein blotting analysis. Relevant experimental steps were performed according to standard protocols. Relevant primary antibodies were: anti-AKAP9 (ab237752,Solarbio),anti-ANK3 (27980-1-ap,Solarbio), anti-LYRM7 (GB115266, Proteintech), anti-MBNL1(66837-1-Ig,Solarbio), anti-α-sma (14395-1-ap,Solarbio).anti-ACTIN (GB15001, Proteintech). Antibodies to C6ORF105 could not be obtained, so its protein was not tested. Cells after modeling were treated with pirfenidone, nidanib, NVP.BE2235, AP24534,Lenalidomide,Nilotinib (B2288-100, A8252-25, A8246-5.1, A5467, A4211-100, A8232-250, APExBIO) using Saline was used as a control, and the number of active cells was determined by CellTiter-Glo Luminescent Cell Viability Assay method (G151, Promega, USA) according to the instructions of the kit procedure. All *in vitro* experiments were repeated 3 times.

### Validation of clinical samples

Peripheral blood was collected from 14 IPF patients in our hospital from January to December 2022, and 10 healthy volunteers were recruited. All participants signed an informed consent form and were approved by the Ethics Committee of the Affiliated Hospital of Shandong University of Traditional Chinese Medicine. Since the annotation of the *NPCDR1* gene was canceled, we examined the mRNA expression levels of *AKAP9, ANK3, C6orf106, LYRM7*, and *MBNL1* genes in the peripheral blood of 14 IPF patients and ten healthy individuals by qRT-PCR. And 3 normal samples and 3 disease samples were randomly selected for protein blotting analysis of key genes, C6ORF105 Reliable antibodies could not be obtained, so they were not determined. Relevant antibody information is as follows: anti-AKAP9 (30290-1-AP, Proteintech Group), anti-ANK3 (27980-1-AP, Proteintech Group), anti-LYRM7 (ab151089, Abcam), anti-MBNL1 (66837-1-Ig. Proteintech Group), anti-actin (GB11001, Servicebio), and experiments were performed according to standard protocols.

The diagnostic effects of these five gene markers were then analyzed using the “pROC” (Version 1.18.0) (31) package, and receiver operating characteristic (ROC) curves were plotted using “ggplot2” (Version 3.3.6).

Total RNA was extracted using the RNAExpress Total RNA Kit (G3013, Servicebio, China). The RNA was reverse transcribed using the RT first strand cDNA Synthesis Kit. We used 2× SYBR Green qPCR Master Mix (None ROX) (Servicebio, China) for RT-qPCR using Bio-Rad CFX96 (Applied Biosystems, USA). These primers are listed in **Table 1**.

**Table 1:** The primer sequences of cuproptosis regulated genes.

Gene	Sequence (5′–3′)	
	Forward	Reverse
GAPDH	GGAAGCTTGTCATCAATGGAAATC	TGATGACCCTTTTGGCTCCC
AKAP9	AGAGTGAGAAACCAAGCCAAGA	CTTCAGTTCAGCAACCACCATT
LYRM7	AAGAAAATAGAAGAGAACTGGTCCC	ATATGCCAGTTCAGGGAAAATGTC
ANK3	ACAACCAATGTTTCAGCCAGAT	CATCGCAAGGAAGATTCTACGG
MBNL1	CAGTTGGAGATAAATGGACGCAA	TGGAGAAACAGGTCCCAGATAG
C6orf105	GTCCTAGATACTGTCATCCCCGTGT	CATAGGATGCGGCTGATGTAAG

### Construction of the nomogram model

We used the “RMS” (Version 6.2.0) software package to create IPF prognostic column line graphs for further informing the clinical prognosis of the screened gene markers and cuproptosis scores. Calibration and decision curve analysis (DCA) were used to estimate the predictive power of the column line graphs.

### Statistical analysis

All statistical analyses were performed using R software 4.1.1. Wilcoxon or t-test was used to analyze the differences between the two groups of variables. Correlations between the variables were determined using Pearson’s or Spearman’s correlation tests. Univariate cox regression analysis and multifactor cox analysis were used to assess the prognostic factors, and hazard ratios (HR) were calculated with a confidence interval (CI) of 95%. All statistical tests were two-tailed, and *p*<0.05 was considered statistically significant.

## Results

### Identification of prognostic features of cuproptosis-related genes

**Figure 1:**
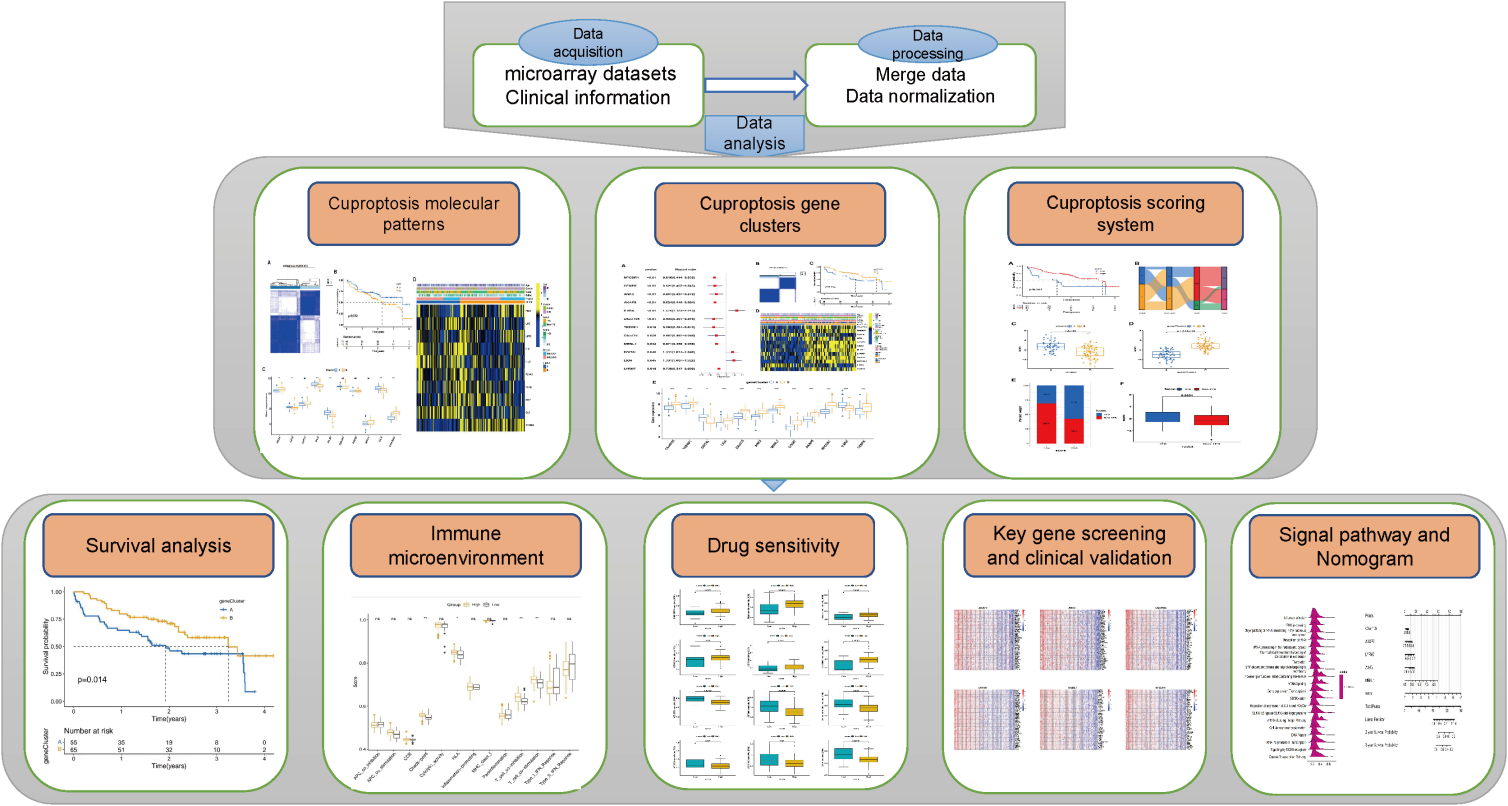
Flow chart.

**Figure 2:**
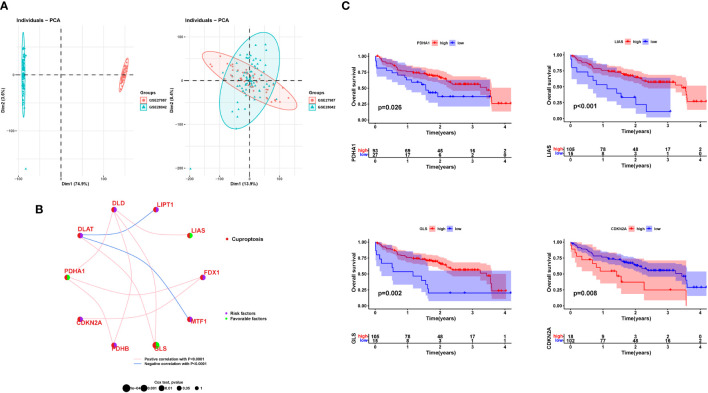
Prognostic features of cuproptosis gene. **(A)** PCA diagram before and after data merging. **(B)** Results of correlation analysis between genes and univariate regression analysis of genes. **(C)** K-M survival curve for each gene. PCA, principal component analysis; K-M, Kaplan–Meier.

At first, patients with IPF in the two datasets, GSE27957 and GSE28042, were combined and analyzed according to **Figure 1**. Treatment was done to remove batch effects so that the two cohorts of patients could be fused for subsequent analysis (**Figure 2A**). Correlation and univariate regression analyses were then performed among ten copper death genes (**Figure 2B**). The results showed that *PDHA1, LIAS, and GLS* were prognostic protective factors, *FDX1, LIPT1, DLD, DLAT, PDHB, MTF1, and CDKN2A* were associated with poor prognosis, and there were significant correlations among the ten genes. Survival analysis showed that the K-M survival curves of four genes (*PDHA1, LIAS, GLS, CDKN2A*) had prognostic significance (**Figure 2C**). These preliminary observations suggested that cuproptosis-related genes may be essential to study in IPF.

### Clinical value of cuproptosis-related molecular patterns in IPF patients

**Figure 3:**
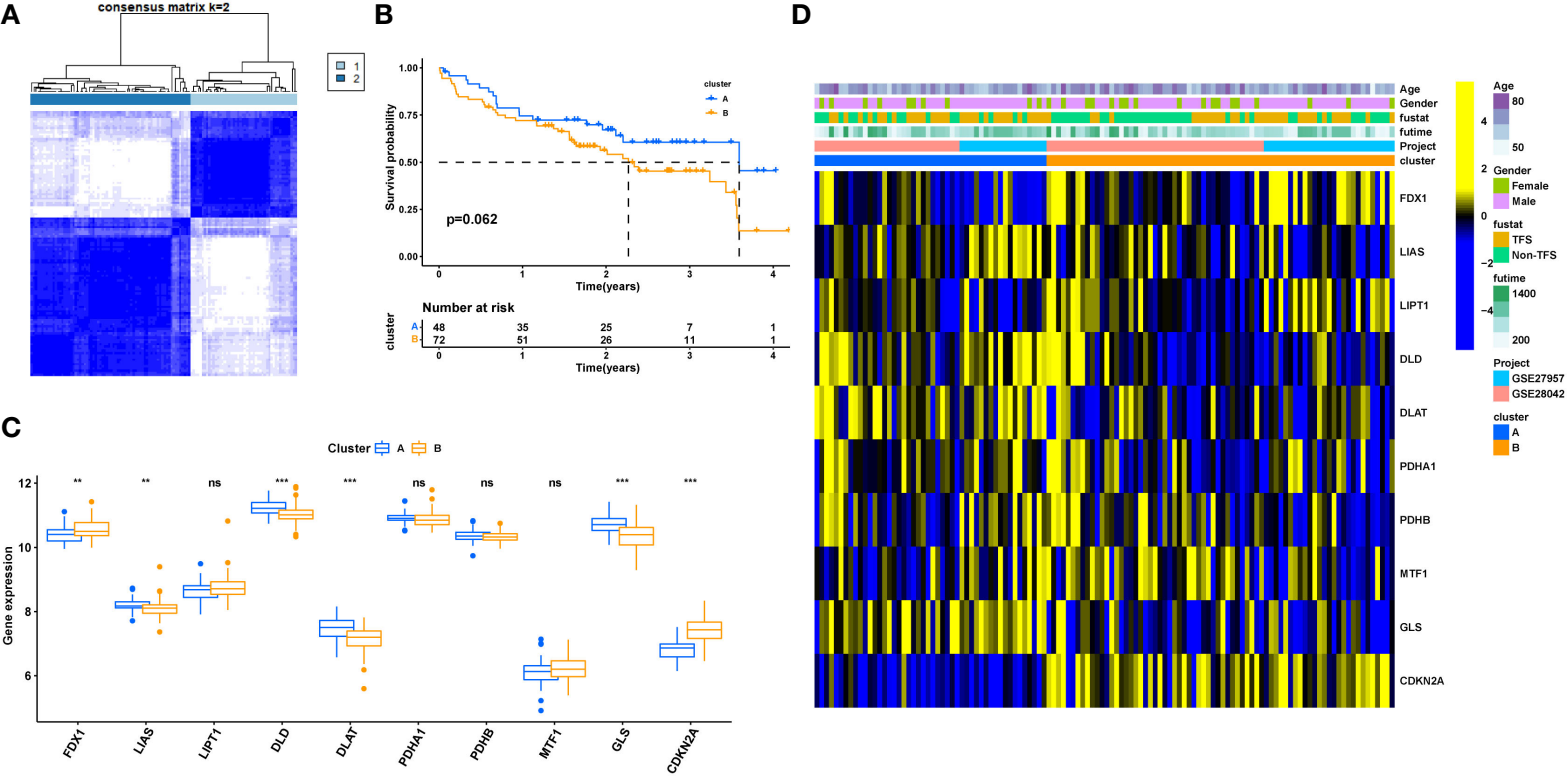
Clinical value of cuproptosis molecular patterns in IPF patients. **(A)** The consensus clustering of cuproptosis genes. **(B)** K-M survival analysis. **(C)** Differential expression of cuproptosis genes between different clusters. **(D)** Relationship between clinical features and clusters. IPF, Idiopathic pulmonary fibrosis; Cluster A= Cluster 1, Cluster B= Cluster 2; TFS, Transplant-free survival; *P < 0.05; **P < 0.01; ***P < 0.001; ns, No statistical significance.

Based on the expression of ten cuproptosis-related genes, an unsupervised clustering algorithm was used to classify 120 IPF patients into two molecular patterns (CluserA, n=48; ClusterB, n=72; **Figure 3A**). The two cuproptosis molecular patterns had different prognostic trends, and patients with ClusterA type appeared to have longer survival times than the other cluster (**Figure 3B**). Differential gene expression analysis showed *LIAS and GLS* expression to be significantly higher in ClusterA than ClusterB, and *FDX1, MTF1, and CDKN2A* expressions were significantly higher in ClusterB, corroborating the trend of the previous analysis (**Figure 3C**). We also analyzed the heat map of the clinical features between the two different molecular patterns in IPF patients (**Figure 3D**). There is heterogeneity between the clinical features of the two molecular models of cuproptosis.

### Analysis of gene set variation in different cuproptosis-associated molecular patterns

GSVA was used to compare the functional differences between the two cuproptosis-associated molecular patterns. The Hallmark gene sets, KEGG, and Reactome pathway gene sets were downloaded separately from the Msigdb database, and the pathways were scored using the R package “GSVA”. The results showed that MYC Targets V1, protein mane, ribosome, RNA degradation, spliceosome, and ALK mutants bind TKIs, RHOBTB2 GTPase cycle, regulation of pyruvate dehydrogenase (PDH) complex pathway is significantly enriched in ClusterA. At the same time, ClusterB-mediated enriched cascades included FGFR1, FGFR1 ligand binding and activation, acetylcholine neurotransmitter release cycle, NF-kB is activated and signals survival, termination of O-glycan biosynthesis, Notch signaling pathway, VEGF signaling pathway, non-small cell lung cancer, Ether lipid metabolism, Wnt/β-Catenin signaling, and other (**Figures S1A–C**). This suggests that there are also significant differences in the biological functions of the two molecular models.

### Identification and functional enrichment analysis of differential genes based on cuproptosis-associated molecular patterns

**Figure 4:**
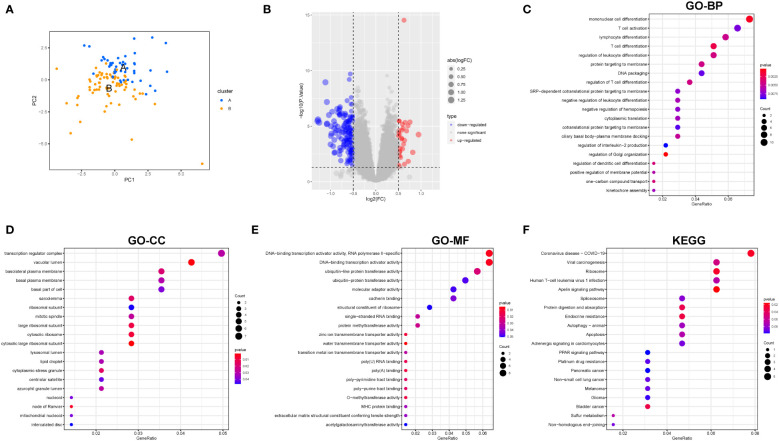
Differential gene expression and functional enrichment analysis among clusterA and clusterB. **(A)** PCA; **(B)** Volcano plot depicted the down- (blue) and upregulated (red) genes between clusterA and clusterB; **(C)** GO-BP analysis; **(D)** GO-CC analysis; **(E)** GO-MF analysis; **(F)** KEGG analysis. BP Biological Process; MF Molecular Function; CC Cellular Component.

**Figure 4A** shows the two types of patients, followed by a differential gene expression analysis of the two cuproptosis-related molecular patterns (clusterA and clusterB), which yielded 178 DEGs, depicted in the volcano plot for the differential analysis (**Figure 4B**). GO analysis found that the regulatory subsets of cuproptosis-related genes were enriched for the terms: T cell activation, lymphocyte differentiation, mononuclear cell differentiation regulation, transcription regulator complex, basolateral plasma membrane, cytosolic ribosome, DNA-binding transcription activator activity, RNA polymerase I-specific, lipid droplet, ubiquitin-like protein transferase activity. In addition, KEGG analysis showed that the genes were involved in Coronavirus disease-COVID-19, viral carcinogenesis, ribosome, apoptosis, Apelin signaling pathway, PPAR signaling pathway, and other signal transduction pathways. (**Figures 4C–F**). GO analysis and KEGG analysis further indicated that cuproptosis-genes are involved in the regulation of a variety of biological activities that are closely related to the development of IPF.

### Clinical value of two cuproptosis-gene clusters in IPF

**Figure 5:**
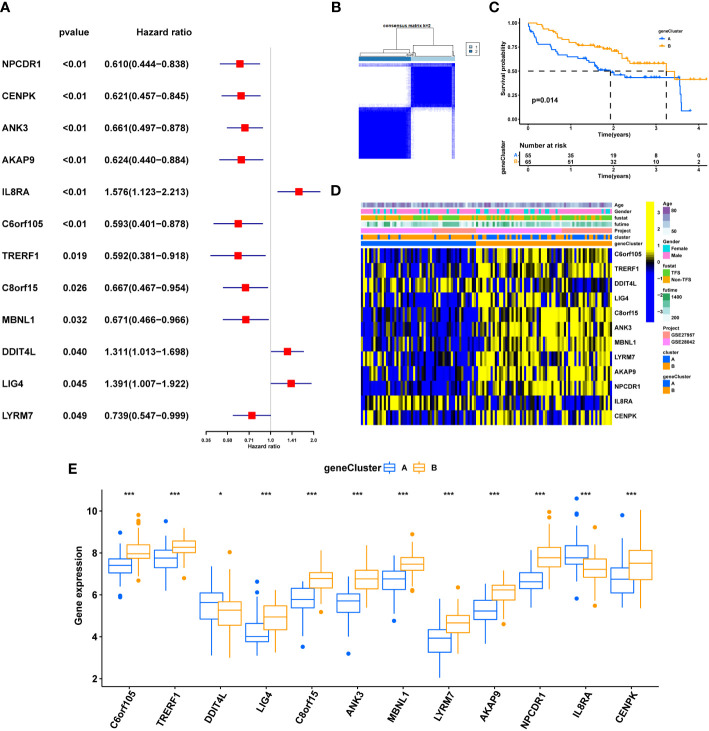
Clinical value of cuproptosis gene clusters. **(A)** Univariate COX analysis of 12 genes with prognostic significance. **(B)** The consensus clustering of Cu-DEGs. **(C)** K-M survival analysis of gene clusters. **(D)** Relationship between clinical features, Gene expression and geneclusters. **(E)** Differential expression of Cu-DEGs between different gene clusters. genecluster A= genecluster 1, genecluster B= genecluster 2; *P < 0.05; **P < 0.01; ***P < 0.001; ns, No statistical significance.

Univariate COX analysis was performed on 178 differential genes (DEGs), and 12 differential genes (Cu-DEGs) with significant correlation (*p*. value < 0.05) with prognosis in IPF patients were screened (**Supplementary Table 1**), *IL8RA, DDIT4L, LIG4* as poor prognosis genes, *C6orf105, TRERF1 C8orf15, ANK3, MBNL1, LYRM7, AKAP9, NPCDR1*, and *CENPK* were associated with prognostic protection in IPF (**Figure 5A**). Unsupervised clustering was used to classify the 120 IPF patients into two clusters (geneClusterA, n=55 and geneClusterB, n=65; **Figure 5B**) based on the expression of 12 Cu-DEGs. K-M survival curves showed that patients in geneClusterB had significantly better survival time than geneClusterA (*p*<0.05) (**Figure 5C**). The characteristics of each clinic showed the differences in Cu-DEGs expression in IPF patients with two gene clusters, corroborating the previous trend of univariate Cox analysis (**Figure 5D**). Interestingly, patients in geneClusterB were also primarily clustered in the cuproptosis molecule pattern ClusterA. **Figure 5E** differential analysis showed that *IL8RA* and *DDIT4L* were significantly more expressed in geneClusterA than in geneClusterB, and *C6orf105, TRERF1, C8orf15, ANK3, MBNL1, LYRM7, AKAP9, NPCDR1, CENPK* in geneClusterB expression was more significant.Re-clustering by differential gene expression was further screened to characterize the differences between the two groups of patients.

### Establishment of a cuproptosis scoring system

**Figure 6:**
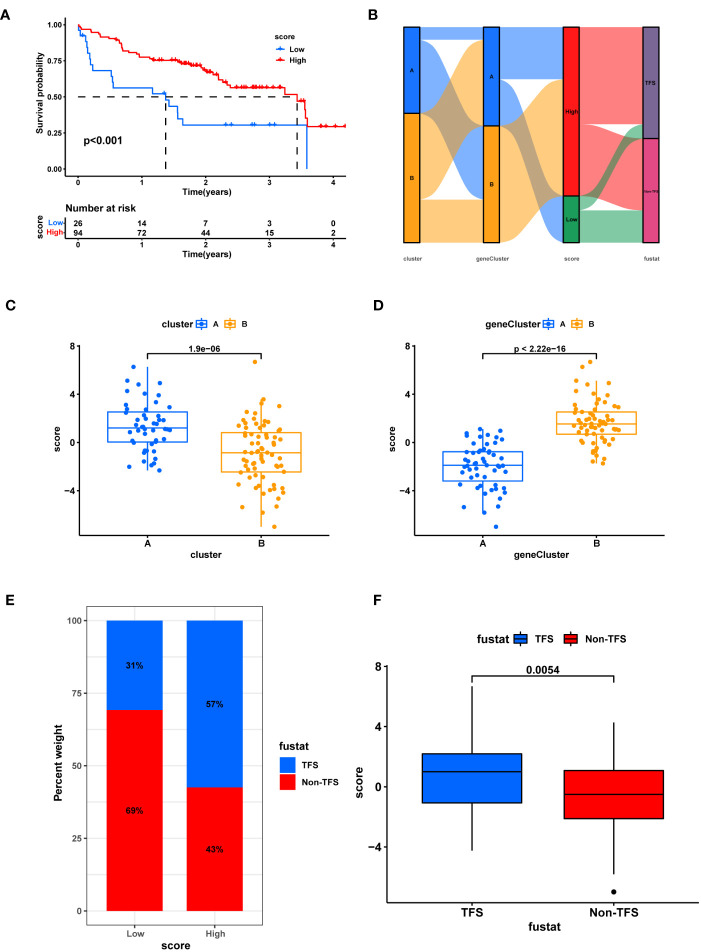
Establishment of cuproptosis scoring system. **(A)** K-M survival analysis between high and low score groups. **(B)** Sankey chart shows the relationship between subtypes, score, and prognostic status. **(C)** Box plot of score difference for cuprortosis clusters. **(D)** Box plot of score difference for cuprortosis geneclusters. **(E)** The ratio of different survival status to high and low score. **(F)** Differences in survival status and score. Non-TFS, Transplanted or death.

**Figure 7:**
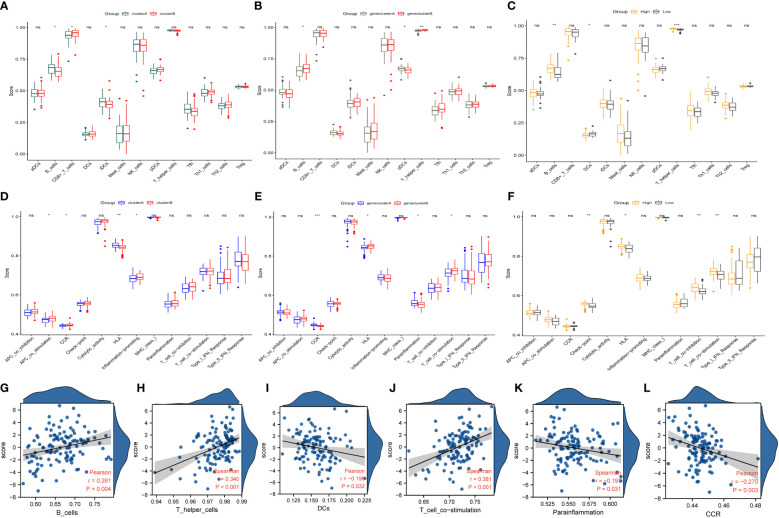
Immune characteristics and correlation between different coproptosis subtypes. **(A, B)** Immunological characteristics of different molecular patterns of coproptosis. **(C, D)** Immune characteristics of different coproptosis geneclusters. **(E, F)** The immune characteristics between different coproptosis score. **(G–L)** Correlations between immune cells, functions, and coproptosis score. HLA, human leukocyte antigen; DCs, dendritic cells; CCR, Cell chemotaxis. *P < 0.05; **P < 0.01; ***P < 0.001; ns, No statistical significance.

The cuproptosis score of each IPF patient was calculated based on principal component (PCA) analysis, and 120 patients were divided into high and low score arrays (score-low, n=26 and score-high, n= 94). The K-M survival curve showed that a higher cuproptosis score significantly correlated with better prognosis of patients (*p*<0.01) (**Figure 6A**). **Figure 6B** shows the differences in the distribution of cuproptosis scores and survival status among IPF patients with different subtypes. Cuproptosis scores were significantly higher for ClusterA compared to ClusterB (*p*=1.9e-06; **Figure 6C**), while geneClusterB had significantly higher cuproptosis scores than geneClusterA (*p*<2.22e-16; **Figure 6D**), consistent with our previous analysis. Intriguingly, the proportion of patients with TFS was as high as 57% among patients with high cuproptosis scores, while only 31% among patients with low cuproptosis scores (**Figure 6E**). **Figure 7F** shows that patients with a survival label defined as TFS had a significantly higher cuproptosis score than those labeled as Non-TFS (p=0.0054), which is of great value. Next, GSEA analysis was performed for both cuproptosis scoring systems. The low-scoring group was associated with neutrophil degranulation, ECM-receptor interaction, and the human complement system as a function (**Figures S2A–C**). The high-scoring group showed significant enrichment of lysine degradation, rRNA processing, T cell receptor signaling pathway, electron transport chain pathway, and other biofunctional pathways (**Figures S2D–G**). Univariate Cox regression analysis showed that gender and cuproptosis score were significantly associated with patient prognosis (**Figure S1H**). Multifactorial Cox regression analysis demonstrated that cuproptosis score was an independent risk factor for IPF (hazard ratio [HR]: 0.834, 95% confidence interval [CI]: 0.747- 0.931, p = 0.001; **Figure S1I**). The ROC curve indicated that the cuproptosis score had good diagnostic efficacy (AUC=0.751, CI: 0.665-0.837. **Figure S4A**). High scores may mean better prognosis for IPF patients.

### Immunological characteristics of different subgroups of IPF and the relationship between immunity and cuproptosis score

The analysis of immune cells and immune function in different clusters of copper death to identify differences in immune cells and immune function among different subtypes showed that: among the molecular patterns regulated by the ten cuproptosis-related genes, clusterA scored higher in B cells, iDCs, and HLA, while clusterB scored higher in CD8+ T cells and functions such as APC co-stimulation, CCR, and Inflammation-promoting (**Figures 7A, B**). Among the gene clusters regulated by the 12 differentially expressed genes, geneClusterA was significantly enriched in pDCs and immune functions such as CCR and Parainflammation. In contrast, geneClusterB was enriched considerably in immune cells such as B cells and T helper cells and processes such as HLA and T cell co-stimulation (**Figures 7C, D**). In the high-scoring group, immune cells such as B cells, T helper cells, and check-point, HLA, T cell co-inhibition, T cell co-stimulation were significantly enriched. In the low-scoring group, DCs showed higher enrichment (**Figures 7E, F**). Interestingly, this distribution of immune characteristics among different clusters of cuproptosis was broadly consistent with the trends in clinical and prognostic markers distribution. Next, we analyzed the correlation between cuproptosis score and immune cells and function. The results showed that the cuproptosis score was significantly positively correlated with B cells, T helper cells, and T cell co-stimulation. At the same time, it was significantly negatively correlated with DCs, parainflammation, and CCR (**Figures 7G–L**). IPF is a highly heterogeneous disease, and immune analyses helped uncover different immune characteristics among other clusters of cuproptosis.

### Validation of external datasets

**Figure 8:**
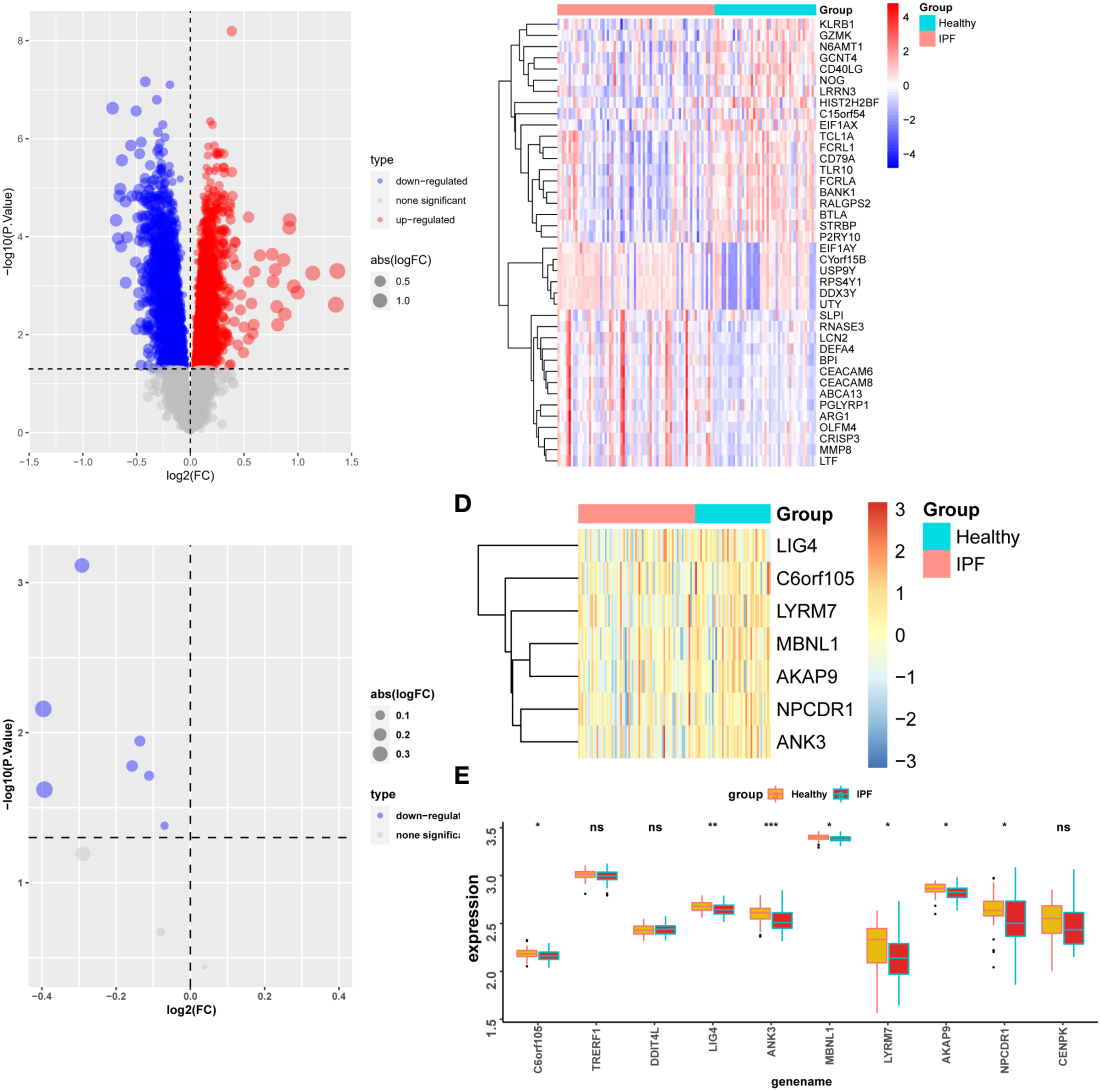
Gene validation in GSE38958 dataset. **(A)** Differential analysis volcano map for the GSE38958 dataset. **(B)** Heatmap of differential gene expression. **(C)** Volcano map of seven differentially expressed genes in the CSE38958 dataset. **(D)** Heatmap of seven differentially expressed genes. **(E)** Box plot of differential expression of seven genes. *P < 0.05; **P < 0.01; ***P < 0.001; ns, No statistical significance.

Differential analysis was performed in the GSE38958 dataset, which revealed that the top 40 genes with differential expression in the healthy population and IPF patients were displayed (**Figures 8A, B**). Twelve differentially expressed genes (Cu-DEGs) obtained from the previous analysis were also validated for differential expression in the GSE38958 dataset, and seven genes with differential expression were obtained and displayed (**Figures 8C, D**). **Figure 8E** shows the differential expression analysis, which revealed the expression of genes such as *C6orf105, LIG4, ANK3, MBNL1, LYRM7, AKAP9*, and *NPCDR1* were significantly higher in the healthy control patients than in the IPF patients. In this analysis, genes associated with IPF were initially identified.

### Screening and functional characterization of key genes

**Figure 9:**
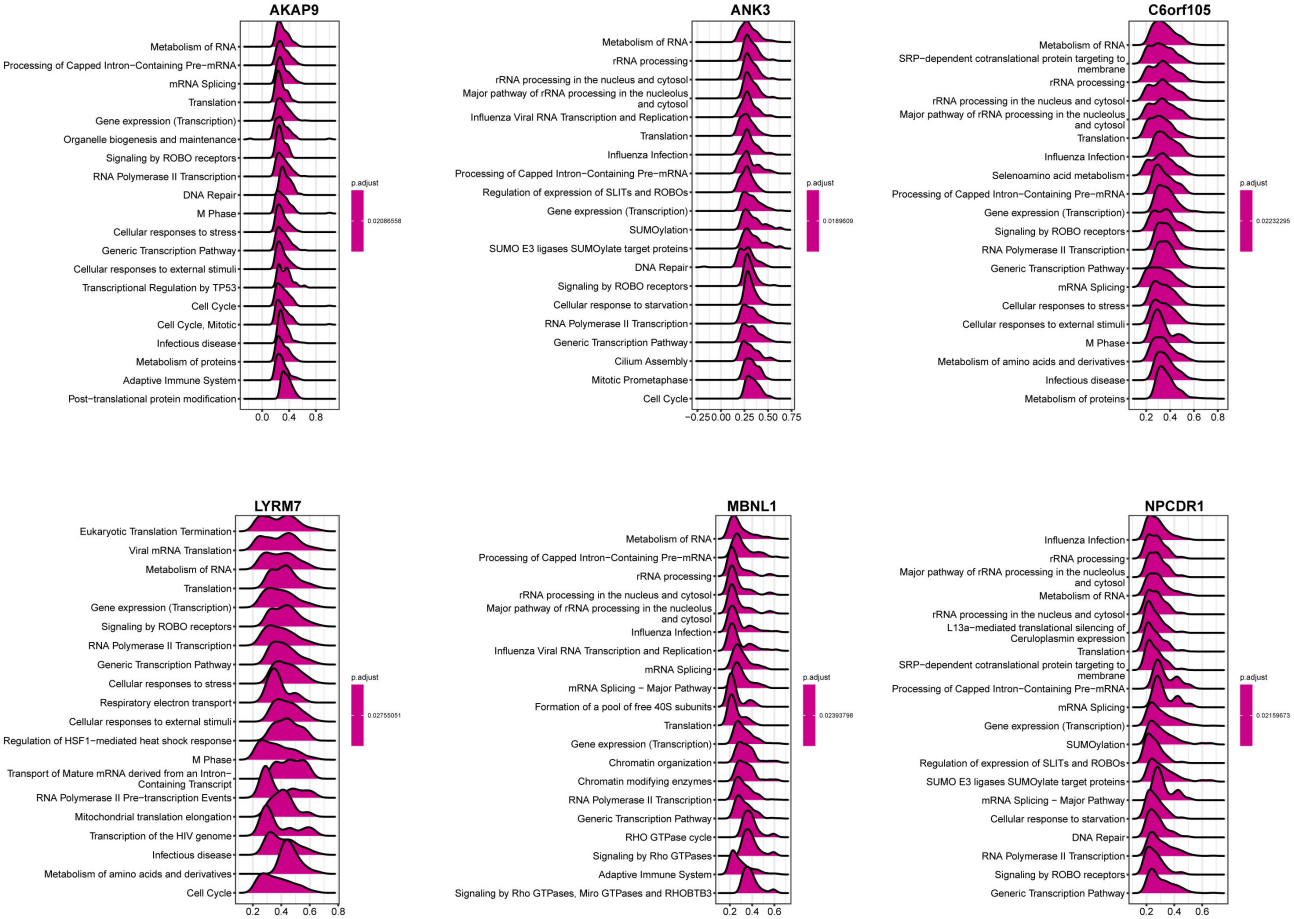
GSEA analysis of single genes. The value of the abscissa represents enrichment analysis. Greater than 0 indicates positive correlation between genes and pathways, while less than 0 indicates negative correlation.

Among the seven genes obtained above, the expression of six was consistent with prognosis according to the multifactorial Cox analysis in **Figure 5A** (*C6orf105, ANK3, MBNL1, LYRM7, AKAP9, NPCDR1*). We defined these six genes as critical for the prognostic assessment of IPF. Subsequently, we performed correlation analysis for each of the six essential genes to show the expression of the 50 genes most positively associated with each gene, and the results showed a significant association between these six genes and other genes (**Figure S3**), indirectly indicating that the six essential genes have a significant impact in IPF. Single-gene GSEA of these six genes was performed, and the results of **Figure 9** show that: AKAP9 gene in the adaptive immune system, cellular responses to external stimuli, transcriptional regulation by TP53, RNA Polymerase II transcription, and other pathways were significantly enriched. ANK3 gene and SUMO E3 ligases SUMOylate target proteins, RNA Polymerase II transcription, metabolism of RNA and rRNA processing, etc., showed a significant positive correlation. C6orf105 gene and metabolism of RNA, gene expression (transcription), SRP-dependent cotranslational protein targeting to membrane, Major pathways of rRNA processing in the nucleolus and cytosol were significantly correlated. LYRM7 gene showed a significant correlation with the metabolism of RNA, cellular responses to stress, gene expression (transcription), and other functions of transport of mature mRNA derived from an intra-containing transcript. MBNL1 and metabolism of RNA, processing of capped intron-containing pre-mRNA, rRNA processing, signaling by Rho GTPases, miro GTPases, and RHOBTB3 are significantly correlated. NPCDR1 gene and L13a-mediated translational silencing to Ceruloplasmin expression, Metabolism of RNA, Major pathways of rRNA processing in the nucleolus and cytosol were significantly correlated. Consistently, the six essential genes were significantly enriched in ribonucleic acid metabolism, transcriptional modifications of mRNA, and protein translation. Key genes are consistent across biogenesis pathways.

### Drug sensitivity analysis based on cuproptosis score

**Figure 10:**
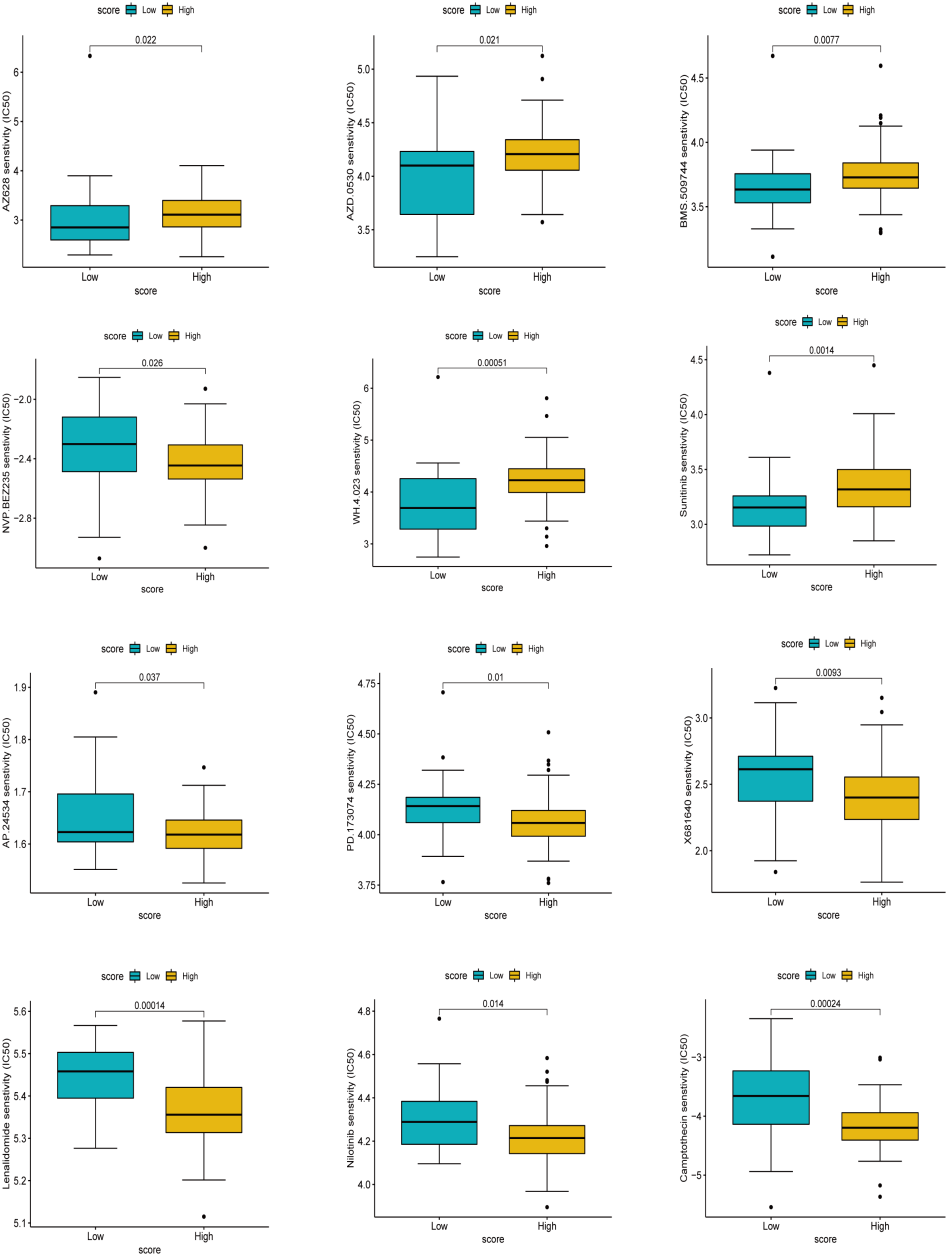
Drug sensitivity analysis based on score. The higher the IC_50_, the less sensitive the treatment.

Extracellular matrix deposition, abnormal neovascularization, fibroblast-myofibroblast transformation, and inflammatory stimuli are closely associated with pulmonary fibrosis (5, 7). We screened drug targets related to the pathogenesis of pulmonary fibrosis among 138 chemical drugs for sensitivity analysis and identified 12 drugs, including AZ628, AZD.0530, BMS.509744, and NVP.BEZ235, Sunitinib, WH.4.023, AP.24534, Camptothecin, Lenalidomide, Nilotinib, PD.173074, and X681640, causing significant clinical differences. The data showed six drugs, AZ628, AZD.0530, BMS.509744, and NVP.BEZ235, Sunitinib, and WH.4.023 had significantly higher half-inhibitory concentrations in the high cuproptosis score group than in the low score group, suggesting that the latter was more sensitive to these six drugs. AP.24534, Camptothecin, Lenalidomide, Nilotinib, PD.173074, and X681640 had significantly higher half-inhibitory concentrations in the low cuproptosis score group than in the high score group; stated differently, patients in the high cuproptosis score group were more sensitive to these drugs (**Figure 10**). Currently, these drugs are mainly used in the antineoplastic field, and their role in treating pulmonary fibrosis remains to be investigated. In fact, some anti-fibrotic drugs, such as nintedanib, were screened precisely among the anti-tumor drugs (36). It is worth mentioning that the treatment of oncology patients is at risk of concomitant interstitial pneumonia (37, 38), and the sensitivity analysis of these drugs also provides a strategy for the treatment of such patients. In order to validate the effect of drug candidates on IPF, we screened four drugs for *in vitro* validation, NVP.BEZ235, AP.24534, Lenalidomide, and Nilotinib, among the above 12 drugs, which had undergone phase II clinical trials and had better toxicological data and were easily accessible. Two conventional therapeutic drugs, Pirfenidone well Nidanib, were used as controls. Cell activity assay showed that at 1um concentration, Lenalidomide, Nilotinib inhibited significantly more than pirfenidone and nidanib, although AP.24534 had similar inhibition at 1um concentration, but did not show more advantageous inhibition at higher concentration (**Figure S6**). However, drug testing between high and low scoring groups has limitations in *in vitro* experiments and should be implemented in future clinical trials by design. In conclusion, we preliminarily explored drugs that are sensitive to IPF treatment, and Lenalidomide, Nilotinib showed better inhibition.

### Correlation analysis of key genes and immune-related factors

**Figure 11:**
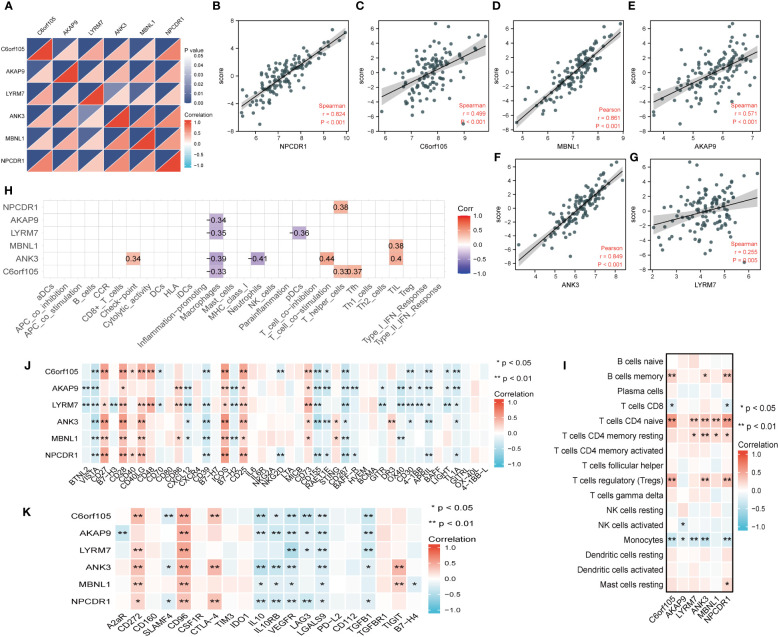
Correlation analysis of key genes with coproptosis score and immune microenvironment. **(A)** Correlations between key genes. The color depth of the lower triangle represents the magnitude of the correlation coefficient, and the upper triangle represents the P-value of the statistical analysis. **(B–G)** Plot of correlation between key gene and coproptosis score. **(H)** Correlation analysis of key genes with immune cells and immune function obtained by ssGSEA algorithm. **(I)** Correlation analysis of key genes with 16 immune cells obtained by cibersort algorithm. **(J)** Heatmap of correlation between key genes and immune stimulators. **(K)** Heatmap of correlation between key genes and immune inhibitors. *P < 0.05; **P < 0.01; ***P < 0.001.

We found a significant positive correlation between the six essential genes (**Figure 11A**). Meanwhile, we tested the correlation between the six key genes and cuproptosis scores according to their expression. We found that all six key genes significantly and positively correlated with the cuproptosis score (**Figures 11B–G**), a trend that confirmed the previous series of analyses. We further performed a correlation analysis of the six critical genes with immune cells and immune functions. As shown in **Figure 11H**, the six crucial genes correlated with various immune cells and functions. We then used 16 immune cell types obtained by the CiberSort algorithm to show the correlations to perform a detailed analysis of the immune cells dictating the expression of the essential genes. The results showed that the vital genes had significant positive correlations with CD4 naïve T cells, CD4 resting memory T cells, and memory B cells and significant negative correlations with activated NK cells and monocytes (**Figure 11I**). To further explore the role of immunomodulation and key genes in IPF, we investigated the correlation between immunostimulatory and immunosuppressive factors. The results showed that CD28, ICOS, CD25, and CD73 were positively correlated with the expression of critical genes, and VISTA and CD267 were negatively correlated with the expression of crucial genes; in other words, the negatively correlated factors were associated with poor prognosis in IPF (**Figure 11J**). **Figure 11K** shows that immunosuppressive factors such as IL10, IL10RB, VEGFR, TGF-β1, and LGASL9 were negatively correlated with critical genes, and CD96 was positively correlated with IPF prognosis. Immunomodulation has an important role in the development of IPF, and analyses have also shown that inflammatory factors are associated with IPF progression, and that immune imbalance is responsible for IPF progression, and further studies still need to be explored at the single-cell level.

### Validation of clinical samples

**Figure 12:**
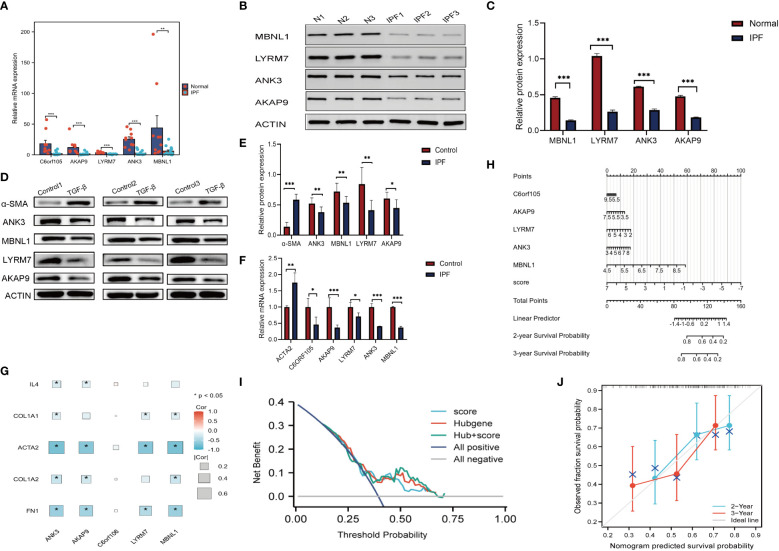
Validation of clinical samples and Nomogram. **(A)** PCR. Relative mRNA expression of key genes in clinical samples. **(B, C)** Relative protein expression of key genes in clinical samples. **(D, E)** Relative protein expression of key genes in MRC-5. **(F)** Relative mRNA expression of key genes in MRC-5. **(G)** Heatmap of key genes in relation to cytokines,extracellular matrix and other fibrosis marker genes. **(H)** Nomogram. **(I)** DCA analysis. **(J)** Calibration chart. *P < 0.05; **P < 0.01; ***P < 0.001.

Single-cell level analysis showed that fibroblast activation was prominent in IPF, and subcellular localization of key genes also showed that five gene markers in IPF lung tissue were more prominently expressed in fibroblasts (**Figure S5**). Therefore subsequent *in vitro* experiments were validated in fibroblasts. We performed quantitative polymerase chain reaction experiments in 14 IPF specimens and 10 normal specimens to verify the bioinformatic results, which showed that genes such as C6orf105, ANK3, MBNL1, LYRM7, and AKAP9 were significantly higher in normal samples than in IPF samples, with the most significant differences in the expression being of ANK3 and LYRM7 (**Figure 12A**). Protein imprinting results were the same as qPCR results (**Figures 12B, C**). *In vitro* experiments, qPCR and protein blotting experiments similarly validated the bioinformatics results (**Figures 12D–F**). Comprehensive data analysis showed that LYRM7 showed the most significant difference in expression. To further explore the relationship between the five key genes and the pathogenesis of pulmonary fibrosis, we correlated the fibrosis marker genes with the key genes, and the results showed that the key genes were closely related to the extracellular matrix genes, and in particular were strongly correlated with the genes, such as ACTA2 and FN1, suggesting that there may be a potential link between the key genes of cuproptosis and the fibroblast-myofibroblast transformation, which also verified the results of the GSEA in **Figure S2** (**Figure 12G**). Diagnostic ROC analysis on these five genes, as shown in (**Figure S4B–F**), showed five genes with diagnostic value. Therefore, we used these genes as prognostic markers. We then constructed a nomogram to predict the survival of IPF patients based on five gene markers and the cuproptosis score to make the cuproptosis-related model more widely available for clinical practice (**Figure 12H**). The accuracy and clinical utility of the combined score and prediction model were determined by 2- and 3-year calibration curves (**Figure 12J**). 2-year decision curve (DCA) analysis showed that the cuproptosis score and the gene marker model had good clinical value. (**Figure 12I**). Clinical samples and *in vitro* experiments verified the reliability of the key genes and that LYRM7 had high differential expression.

## Discussion

In this study, we constructed three cuproptosis subtypes in IPF and performed a comprehensive analysis of the patients’ clinical, immunological, and prognostic characteristics. This task proposes using a cuproptosis score to quantify the different subtyping patterns in IPF patients. We also propose to assess the prognostic characteristics of patients. We further screened reliable marker genes for predicting disease prognosis. We conducted an in-depth analysis of the immune regulatory pathways associated with the expression of the marker genes to improve the understanding of immune regulation mechanisms in IPF. The study further localized fibroblast activation at the single-cell level as an important mechanism of IPF genesis. Finally, we established a column line plot of the cuproptosis score with five marker genes, hypothesizing they might be a more reliable reference for clinicians in the prognostic diagnosis and treatment of IPF.

Cuproptosis is a recently identified and regulated form of copper ion-dependent cell death. The pathogenesis of IPF is complex and unclear, with numerous associations with copper ions (17–19). We performed prognostic characterization of cuproptosis-related genes based on their expression levels in the IPF gene expression matrix. Initially, we demonstrated a correlation between cuproptosis-related genes and the prognosis of IPF at the genomic level. Next, we classified patients into two subtypes depending on their molecular patterns of cuproptosis based on cuproptosis-related regulatory gene expression. The two patterns differed in prognosis, clinical features, and biological functions. Patients in clusterA were mainly correlated to MYC targets V1, protein fascinators, ribosome, RNA degradation, spliceosome, RHOBTB2 GTPase Cycle, regulation of pyruvate dehydrogenase (PDH) complex, cluster B was significantly enriched in FGFR1 ligand binding and activation pathways, SHC-mediated cascade: FGFR1, NF-kB is activated and signals survival, termination of O-glycan biosynthesis, Notch signaling pathway, VEGF signaling pathway, non-small cell lung cancer, ether lipid metabolism and Wnt/β-Catenin signaling. TGF-β1 has been shown to partially regulate fibroblast activation by reducing acetyl coenzyme A synthesis through its effect on pyruvate-dependent PDHc activity, and TGF-β1 plays a crucial role in epithelial-mesenchymal transition (EMT) and fibrogenesis (39, 40). RHOBTB2 GTPase is associated with CXCL14, which controls inflammation and angiogenesis, while a hypoxic environment is closely related to CXC chemokines. Related studies also confirmed CXCL14 to be a positive metastable regulator of CXCR4 that acts synergistically with CXCL12 in different cellular responses. Unfortunately, CXCL14 was not detected in the datasets of this study, which may arise due to sample processing. However, the CXCL12-CXCR4-CXCL14 regulatory model is still not clearly defined in IPF (41–43). FGFR, activated NF-kB and VEGF are potent fibroblast mitogens that play essential roles in the proliferation of myofibroblasts (44, 45); these observations collectively indicated that cuproptosis might be involved in these aspects of the development of IPF.

Alterations in gene levels, transcriptional processes, and protein modifications likewise have an essential role in the pathogenesis of the IPF (46–48). The functional analysis of differential genes between the two different molecular pattern groups revealed significant involvement of immune responses, regulation of cell cycle life activities such as gene transcription and ribosomes, and fatty acid metabolism. Such molecular patterns are also associated with viral diseases and non-small cell lung cancer. Next, we developed two gene clusters with different clinical characteristics, prognostic regression by cuproptosis differential genes (Cu-DEGs). We used the PCA algorithm to score cuproptosis for each patient, and clusterA and geneclusterB obtained relatively high cuproptosis scores. K-M survival analysis showed that patients with high cuproptosis scores had better prognoses, suggesting that low cuproptosis scores contribute to poor prognoses in IPF. Cuproptosis is closely related to the electron transport chain complex and protein lipoylation. Accordingly, GSEA showed the high-scoring group associated with lysine degradation, rRNA processing, T cell receptor signaling pathway, and electron transport chain pathway. Protein lipoylation is a post-translational modification of lysine, and its metabolism is closely related to copper protein and pulmonary fibrosis diseases (49–51). Consistently, both cuproptosis scoring systems were enriched in immunomodulation-related pathways, suggesting the possibility of cuproptosis regulating immunity. We further analyzed the extent of immune infiltration of the three clusters of cuproptosis, which revealed that the cluster with a poorer prognosis was mainly associated with inflammation promotion. In comparison, the cluster with a better prognosis was associated with B cells, T helper cells, and bidirectional regulation of immunity. It is known that CD8+ T cells in alveolar lavage fluid of IPF patients show a positive correlation with modified British medical research council(MRC) dyspnea grade (52). In contrast, T helper cells are more implicated in bidirectional regulatory roles in the IPF pathogenesis (53).

Next, we combined external validation datasets and cases collected at our institution to find five diagnostic markers (*C6orf105, ANK3, MBNL1, LYRM7, AKAP9*) with positive prognostic effects, which are widely involved in ribosome synthesis and processing, transcription, cytoskeleton regulation, immunity, and other biological functions. This investigation aimed to further search for meaningful markers for prognostic diagnosis of IPF. C6orf105 is an androgen-dependent TFPI regulatory protein that enhances the activity of tissue factor pathway inhibitor (TFPI), which cannot counteract tissue factor TF in broncho alveolar lavage (BAL) of patients with advanced IPF, leaving the lungs of IPF patients in a hypercoagulable state (54, 55). Overexpression of ANK3 leads to enhanced degradation of the platelet-derived factor (PDGFR) (56). The LYRM7 gene is associated with the metabolism of Fe-S cluster proteins and maintains factor stability during the functioning of the mitochondrial respiratory chain complex enzymes (57). AKAP9 complex inhibits collagen levels and promotes the lipid mediator prostaglandin E2 (PGE) anti-fibrotic effect on IPF (58). Recent studies have shown that MBNL1 can dynamically stabilize scar formation in the transformation of fibroblasts to myofibrosis (59). These pieces of evidence suggest that genetic markers may have a role in pulmonary fibrosis. Moreover, the expression of all five marker genes showed a positive correlation with the cuproptosis score. Furthermore, the ROC curve also showed diagnostic value, indicating the reliability of the five marker genes. We further analyzed the relationship between fibrosis marker genes and key genes, and showed that activation of myofibroblasts was significantly correlated with key genes, and that low copper death scoring clusters were enriched in extracellular matrix receptors in the pre-GSEA, suggesting that there may be a potential link between copper death and IPF in this mechanism. Subsequently, these markers and cuproptosis scores were constructed as IPF 2-year and 3-year prognostic column line graphs, and this combined model improved the accuracy of clinical application.

Immunological analysis of cuproptosis-related clusters revealed differences in immunomodulation between different subtypes of IPF. However, specific immunomodulatory factors have still not been explored in depth. We further analyzed the roles of the immune cells based on the expression of the crucial genes. We showed that naïve CD4+ T cells, T cells with resting CD4 memory, and memory B cells were positively correlated with the essential genes. Previous studies showed that these cells could improve the body’s immune surveillance and speed up the immune response (60–62). Huang et al. investigated the relationship between NK cells and FVC decline at the single-cell transcriptome level to confirm the involvement of NK cells in IPF progression (63). In a retrospective study of the relationship between monocyte count stratification and the prognosis of patients with IPF, elevated monocyte counts were associated with an increased risk of IPF progression, hospitalization, and death (64). The literature further corroborated our bioinformatics analysis. We further analyzed the immunosuppressive and immune-activating factors to identify changes in IPF. Interestingly, an earlier study by Herazo-Maya et al. found that the T-cell co-stimulatory proteins ICOS and CD28 and “co-stimulatory signaling during T-cell activation” may predict a shorter TFS in IPF patients (21). Several other studies in a colorectal mouse model showed that VISTA expression was positively correlated with hypoxia (65). However, studies on the functional role of VISTA have been somewhat lacking in exploring lung models. CD96 is an immunosuppressive factor that negatively regulates the responsiveness of NK cell-related factors (66). We hypothesized this could be a possible pathway to avoid immune overexpression in IPF, but no study has confirmed this possibility.

We noted the study of Li et al., which observed the developmental trajectory of fibroblasts at the single-cell level and analyzed the correlation with the expression of cuproptosis gene, and finally concluded that cuproptosis was negatively correlated with pulmonary fibrosis (67). Our study started from the premise that the expression of cuproptosis genes in IPF is heterogeneous, performed three times of typing, and finally carried out the calculation of cuproptosis score with the screening verification of key genes, to target the relationship between cuproptosis and fibroblasts at the transcriptome and single-cell level, and the results of the two studies were consistent with each other, and their methods were complementary to each other.

Many shortcomings remain in this study: first, although we provide novel insights into mechanisms underlying cuproptosis and its immune regulation, specifically related mechanisms regulating cuproptosis-related patterns in IPF remain unexplored; secondly, there are limitations in our application regarding drug sensitivity in copper death scoring, as there is a lack of sufficient data for accurate prediction due to the current drug studies on IPF and the low application of databases. Although the effects of the four drugs were validated in *in vitro* experiments, the relationship between scores and drugs still needs to be carried out in clinical trials.

## Conclusion

In this study, we explored the differences in prognosis, clinical characteristics, and immunity among different clusters associated with cuproptosis and constructed a cuproptosis score to quantify the prognosis of IPF patients. We screened five marker genes associated with prognosis for in-depth analysis of immunological aspects. Finally, we constructed column plots of the cuproptosis score and the marker genes to predict patient survival, forming a reference for clinical treatment decisions.

## Data availability statement

The original contributions presented in the study are included in the article/**Supplementary Material**. Further inquiries can be directed to the corresponding author.

## Ethics statement

The studies involving humans were approved by The Ethics Committee of the Affiliated Hospital of Shandong University of Traditional Chinese Medicine. The studies were conducted in accordance with the local legislation and institutional requirements. The participants provided their written informed consent to participate in this study.

## Author contributions

CJ: Conceptualization, Methodology, Writing – original draft. RF: Formal Analysis, Software, Writing – original draft. XL: Conceptualization, Resources, Supervision, Writing – review & editing. GZ: Data curation, Project administration, Resources, Writing – review & editing. XZ: Data curation, Investigation, Project administration, Writing – review & editing. CW: Formal Analysis, Visualization, Writing – review & editing. WZ: Conceptualization, Funding acquisition, Resources, Supervision, Validation, Writing – review & editing.
