# Supplementary material for: A comprehensive cuproptosis score and associated gene signatures reveal prognostic and immunological features of idiopathic pulmonary fibrosis
Source: Front Immunol. 2023 Nov 14;14:1268141. doi: 10.3389/fimmu.2023.1268141 (PMC10682708; doi:10.3389/fimmu.2023.1268141)
Supplement: Supplementary file 1 [file DataSheet_1.docx]

Supplementary Material

# Supplementary Figures and Tables

## Supplementary Figures


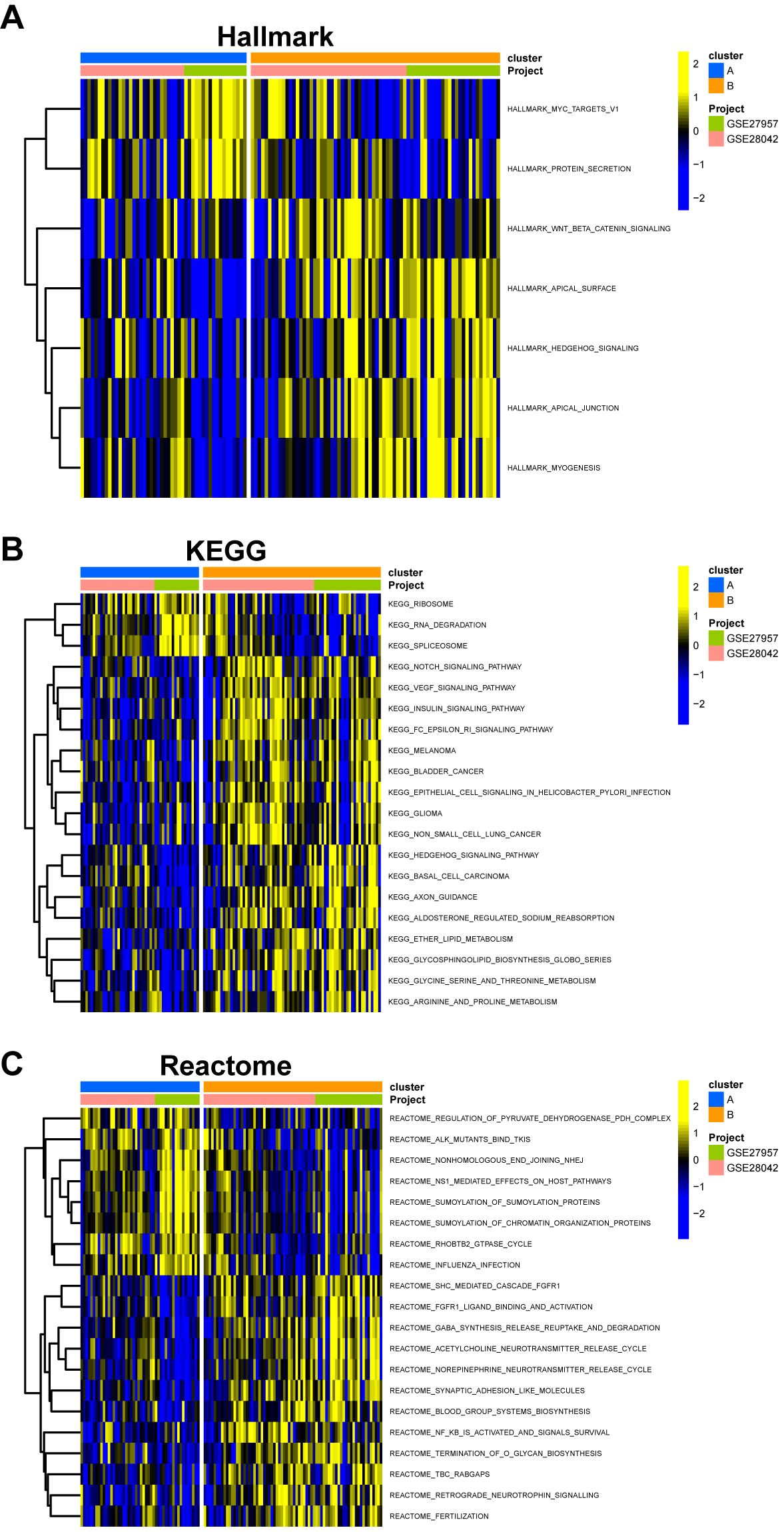


**FIGURE S1 | GSVA with different molecular patterns associated with cuproptosis. (A) HALLMARK; (B) KEGG;(C) Reactome. GSVA, Gene set variation analysis.**


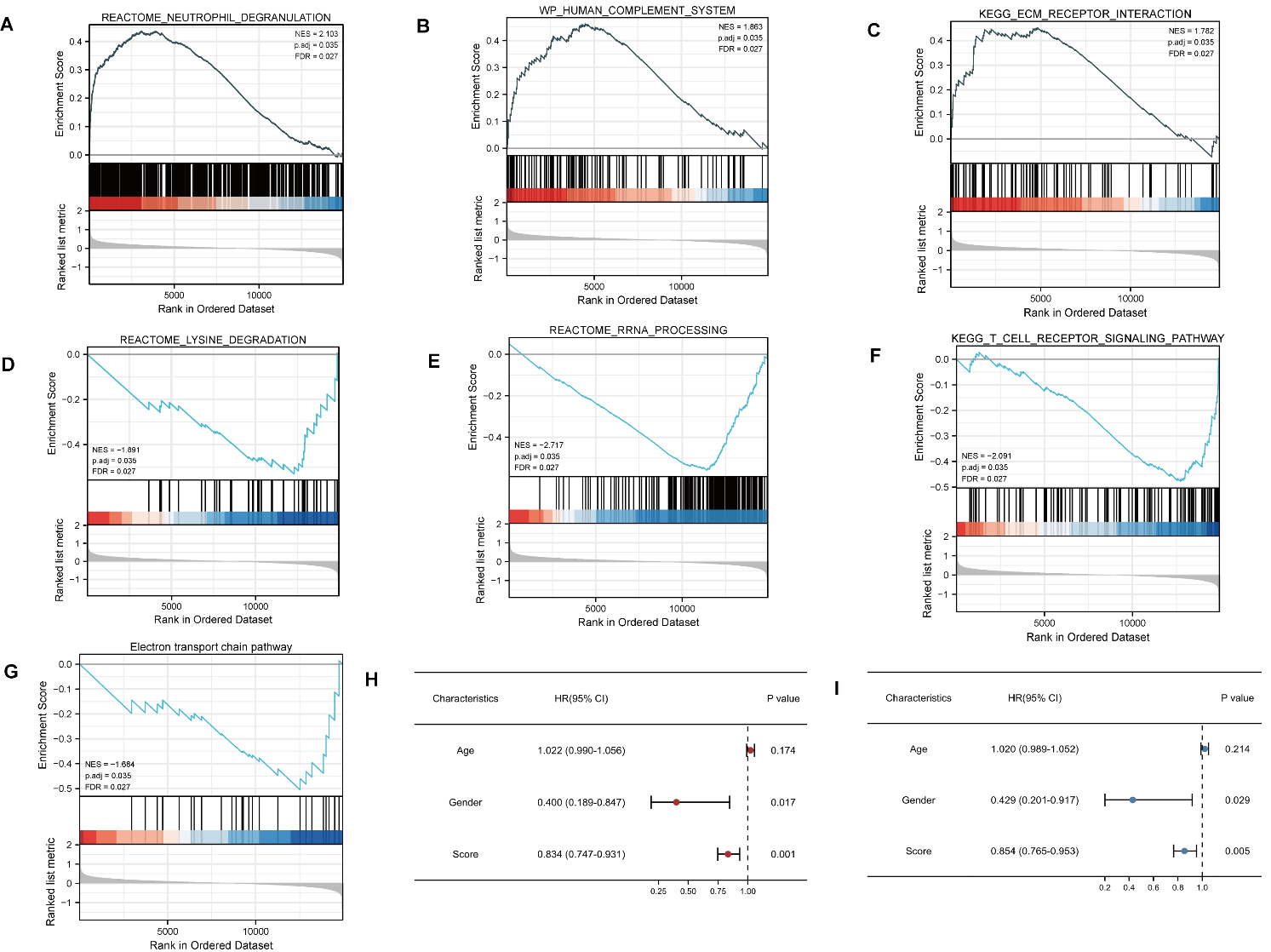


**FIGURE S2 | (A-C) GSEA enrichment analysis of Low-score group. (D-G) GSEA enrichment analysis of High-score group. (H) Univariate cox analysis. (I) Multivariate cox analysis.**


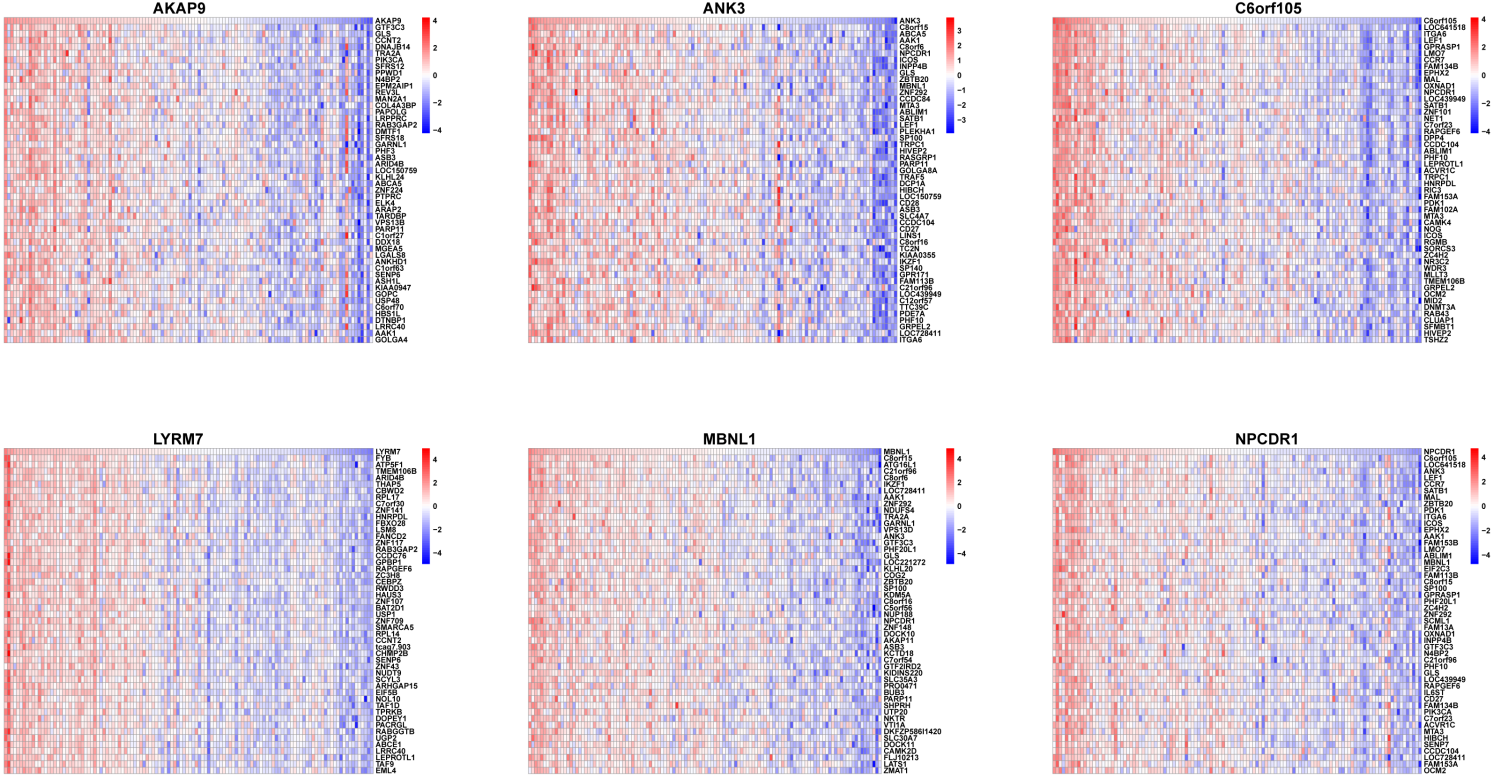


**FIGURE S3 | Correlation analysis of key genes.**


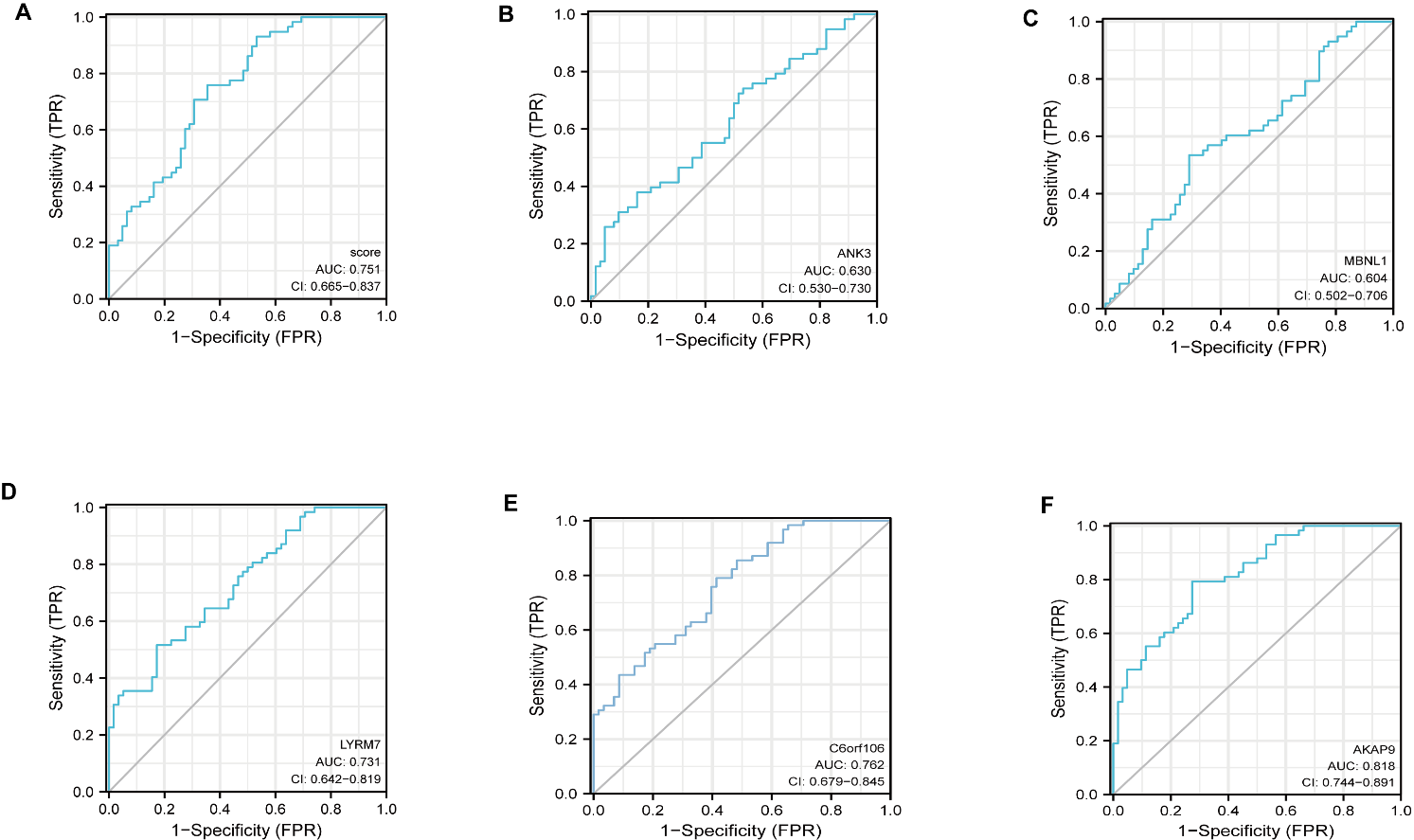


**FIGURE S4 | (A) ROC curve of coproptosis score. (B-F) ROC curves of 5 gene signatures.**

**
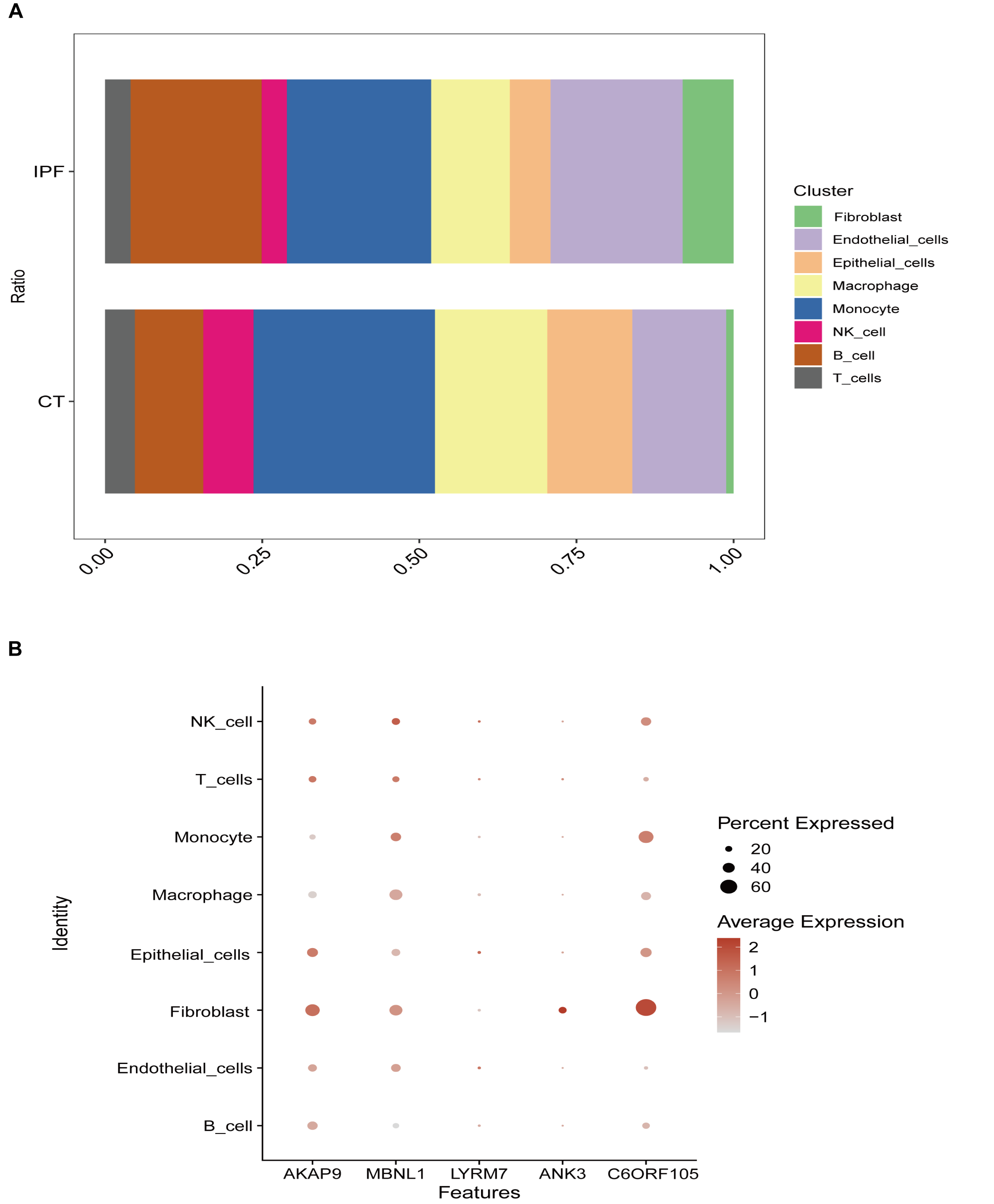
**

**FIGURE S5 | (A) Comparison of cell ratios. (B) Map of subcellular localization of key genes.**

**
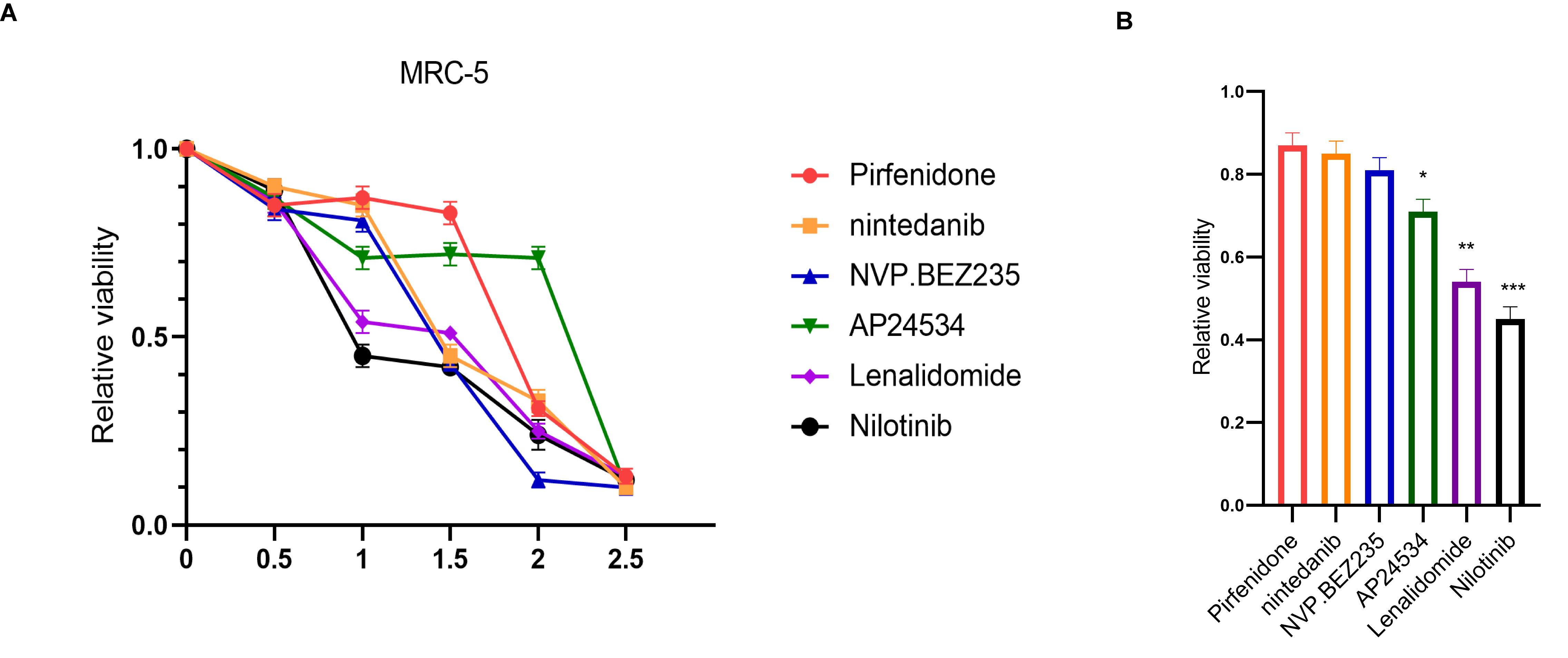
**

**FIGURES6 | (A) Cell survival curves of MRC-5 cells treated with the indicated inhibitors in dose increments from 0 to 3 μM. Data are expressed as mean ± SD.(B) Relative viability in the MRC-5 cell line under drug treatment at a concentration of 1 μM.*P < 0.05; **P < 0.01; ***P < 0.001.**

## Supplementary Table

**Sup-Table1 |** Univariate COX results of Cu-DEG

| **Gene** | **HR** | **HR.95L** | **HR.95H** | **pvalue** |
| --- | --- | --- | --- | --- |
| NPCDR1 | 0.61009 | 0.444035 | 0.838244 | 0.0023 |
| CENPK | 0.62103 | 0.456551 | 0.844765 | 0.002409 |
| ANK3 | 0.660837 | 0.49726 | 0.878225 | 0.004305 |
| AKAP9 | 0.623549 | 0.439923 | 0.883822 | 0.007957 |
| IL8RA | 1.576363 | 1.122917 | 2.212917 | 0.008542 |
| C6orf105 | 0.593355 | 0.401196 | 0.877551 | 0.008945 |
| TRERF1 | 0.591595 | 0.381413 | 0.917599 | 0.019081 |
| C8orf15 | 0.667296 | 0.466839 | 0.953828 | 0.026465 |
| MBNL1 | 0.670525 | 0.465623 | 0.965597 | 0.031705 |
| DDIT4L | 1.311334 | 1.012994 | 1.697538 | 0.039591 |
| LIG4 | 1.39117 | 1.007095 | 1.92172 | 0.045193 |
| LYRM7 | 0.739241 | 0.546969 | 0.9991 | 0.049319 |
